# A novel bifunctional mitochondria-targeted anticancer agent with high selectivity for cancer cells

**DOI:** 10.1038/srep13543

**Published:** 2015-09-04

**Authors:** Huan He, Dong-Wei Li, Li-Yun Yang, Li Fu, Xun-Jin Zhu, Wai-Kwok Wong, Feng-Lei Jiang, Yi Liu

**Affiliations:** 1 State Key Laboratory of Virology & Key Laboratory of Analytical Chemistry for Biology and Medicine (MOE), College of Chemistry Molecular Sciences, Wuhan University, Wuhan 430072, P. R. China; 2 Department of Chemistry, Hong Kong Baptist University, Kowloon Tong, Hong Kong, P. R. China

## Abstract

Mitochondria have recently emerged as novel targets for cancer therapy due to its important roles in fundamental cellular function. Discovery of new chemotherapeutic agents that allow for simultaneous treatment and visualization of cancer is urgent. Herein, we demonstrate a novel bifunctional mitochondria-targeted anticancer agent (FPB), exhibiting both imaging capability and anticancer activity. It can selectively accumulate in mitochondria and induce cell apoptosis. Notably, it results in much higher toxicity toward cancer cells owing to much higher uptake by cancer cells. These features make it highly attractive in cancer imaging and treatment.

Mitochondria play a crucial role in energy producing, programmed cell death regulation, reactive oxygen species (ROS) generation and calcium homeostasis maintenance. Mitochondrial targeted drugs, namely mitocans^1^, represent a new strategy for antitumor therapy due to the significant difference in structure and function of mitochondria between cancer and normal cells^2^. Delocalized lipophilic cations (DLCs) offer a successful approach to mitochondria-targeted chemotherapy for its capacity to selectively target tumour cells^3^. Driven by the higher mitochondrial membrane potential (*ΔΨ*_*m*_)^4^, DLCs such as Rhodamine 123^5^, MKT-077^6^ and F16^7^,^8^ could concentrate in carcinoma mitochondria and induce toxicity to mitochondria. Extensive studies have shown that the 60 mV difference in *ΔΨ*_*m*_ between carcinoma and normal cells is sufficient to account for 10-fold greater accumulation of DLCs in mitochondria^9^,^10^,^11^,^12^. Recent studies suggest that DLCs could act as cargo groups to selectively delivery functional molecules to mitochondria in intact cells^13^,^14^,^15^,^16^,^17^. Among these delivery groups, triphenylphosphine (TPP) is the most widely used and successful mitochondria-targeted delivery moiety^18^,^19^. Besides, F16 is a kind of attractive and promising mitochondria-targeted DLCs that selectively inhibits tumour cell proliferation^7^ but further research and utilization of F16 is rare.

Currently, extensive research has focused on the discovery of new multifunctional anticancer agents which allows for simultaneous monitoring, treatment and direct visualization of cancer^20^,^21^. The ability to see within the living human body and cure the malignancy at the same time is one of the most exciting and rapid growing areas of science. In the previous studies, we reported bifunctional imaging and therapeutic agents by conjugation of a lanthanide complex or quantum dots with a photo-toxic porphyrin^22^,^23^. Moreover, we have synthesized several organic compounds bearing multifunctional moieties for more-application-with-one-molecule^24^,^25^. Despites these interesting results, there is still a compelling necessity for the discovery of new anticancer agent that achieves integrated imaging and therapeutic function, especially those with capability of selective accumulation in cancer cells. Among the various molecular imaging techniques, fluorescence imaging offers the best spatial resolution and exquisite molecular specificity, therefore is one of the most powerful and widely used tools to understand complex biological processes for cells, tissues and bodies in preclinical studies.

We now report a novel bifunctional mitochondria-targeted anticancer agent **FPB**, by conjugating F16, a DLC compound, and boron-dipyrromethene (BODIPY), a widely used fluorescent dye, with a phenylethynyl linker (Fig. 1). To the best of our knowledge, there are few reports on utilizing F16 as a cargo group to target mitochondria. Biological investigations suggest that **FPB** is a promising multifunctional anticancer agent that incorporates optical monitoring capability and selective anticancer activity. **FPB** could accumulate in carcinoma mitochondria and induce cell apoptosis. Moreover, our study suggested that F16 could also be used as a mitochondria-targeted moiety to design anticancer drugs.

## Results

### Synthesis of the FPB

**Figure 1:**
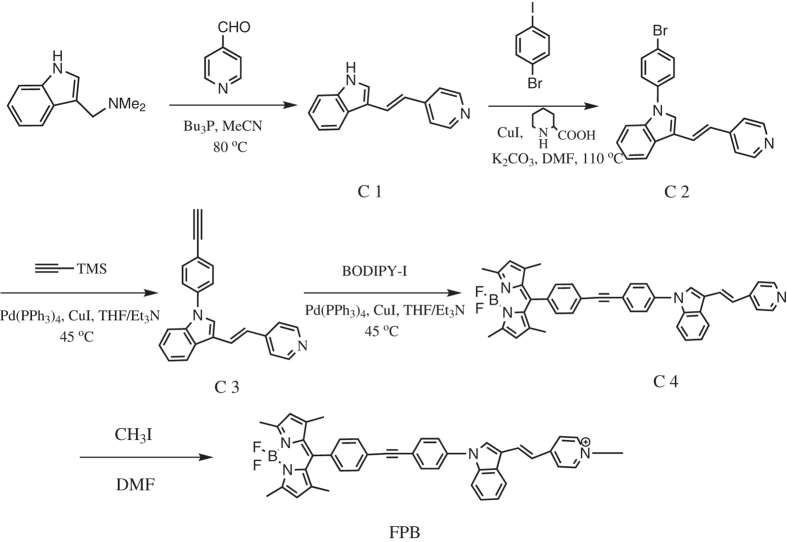
The synthetic route of FPB.

Synthesis of the **FPB** conjugate is shown in Fig. 1. Firstly, reaction of gramine with pyridinecarboxaldehyde in the presence of N-butyl phosphorus affords C1 in in yield of 41%. C1 undergoes CuI-catalyzed Ullmann and Goldberg coupling reaction^26^ with 1-bromo-4-iodobenzene gives C2 in 50% yield. Treatment of C2 with trimethyl silyl acetylene in the presence of CuI, Pd(PPh_3_)_4_, THF and triethylamine gives C3 in 90% yield. BODIPY-I is synthesized according to our previous work^27^ with a little modification (Fig. S1, see Supporting Information (SI)). Reaction of C3 with BODIPY-I in the presence of CuI, Pd(PPh_3_)_4_, THF and triethylamine affords C4 in 33% yield. Methylation of C4 with iodomethane affords **FPB** in 88% yield. For comparison, C3 is also methylated to get F16-Ph-Ace as a reference compound (Fig. S2 (SI)).

### Cytotoxicity and selectivity

**Figure 2:**
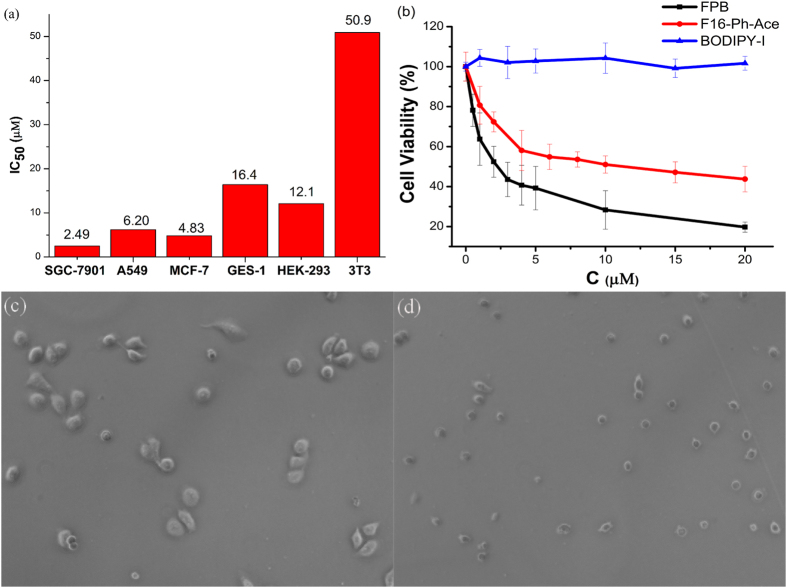
(**a**) *IC*_50_ values of **FPB** for different cell lines. (**b**) Cell viability of SGC-7901 treated with **FPB**, F16-Ph-Ace, and BODIPY-I using a typical MTT assay. (**c**) Phase contrast images of SGC-7901 cell lines treated without **FPB** for 48 h. (**d**) Phase contrast images of SGC-7901 cell lines treated with **FPB** (5 μM) for 48 h.

The cytotoxicity of **FPB** was first studied by the typical MTT [3-(4,5-dimethylthiazol)-2,5-diphenyltetrazolium bromide] method. Three normal cell lines, human gastric mucosal epithelial cell lines (GES-1), human embryonic kidney 293 cell lines (HEK293) and mouse fibroblast cell lines (NIH/3T3), and three cancer cell lines, gastric cancer cell lines (SGC-7901), pulmonary adenocarcinoma cell lines (A549) and human breast adenocarcinoma cell lines (MCF-7) were used as target cells. The cytotoxicity data of **FPB** toward the six different cells are provided in Fig. S5 (SI) and the *IC*_50_ values were calculated as shown in Fig. 2a. Noteworthy, **FPB** shows different toxic effects towards carcinoma and normal cell lines. All carcinoma cell lines (SGC-7901, MCF-7 and A549) are sensitive to **FPB** with the *IC*_50_ in the range of 2.49 μM to 6.20 μM, while two normal cell lines (GES-1 and HEK293) are less sensitive to **FPB** with the *IC*_50_ of 16.4 μM and 12.2 μM. Especially for SGC-7901 and GES-1, the human gastric carcinoma cells and corresponding normal cells, the selective factor (ratio of *IC*_50_) is about 6.58. GES-1 and HEK293 cells are not strictly normal cells as they are transformed with SV40 or Ad5 virus, so we further tested the toxicity of **FPB** on the normal fibroblast cells. The results indicated that the toxicity of **FPB** on 3T3 cells is lower compared with GES-1 and HEK293. The phase contrast images of SGC-7901 cells were also taken before and after incubating with 5 μM **FPB** for 48 h. As shown in Fig. 2c,d, the cell volume of SGC-7901 cells treated with **FPB** were obviously smaller than that without **FPB**. The reduced cell size and abnormal cellular morphology indicated that the cells were dead or in the late stage of apoptosis. This result is consistent with the MTT experiment. These results demonstrate that **FPB** can act as an anticancer agent of broad spectrum and exhibits good selectivity to cancerous cell lines over normal cell lines.

We further investigated the cytotoxicity of BODIPY-I and F16-Ph-Ace for a structure-activity relationship study. As shown in Fig. 2b, F16-Ph-Ace could effectively inhibit SGC-7901 cells growth (*IC*_50_ = 15.1 μM) and BODIPY-I exhibits no obvious toxicity on SGC-7901 cell lines (*IC*_50_ > 100 μM). Surprisingly, conjugation of F16-Ph-Ace and nontoxic BODIPY-I exhibits better anticancer activity efficiency (five folds smaller than F16-Ph-Ace in *IC*_50_). The results suggest that the BODIPY part of **FPB** might play a specific role for its toxicity.

### Effects on cell proliferation

**Figure 3:**
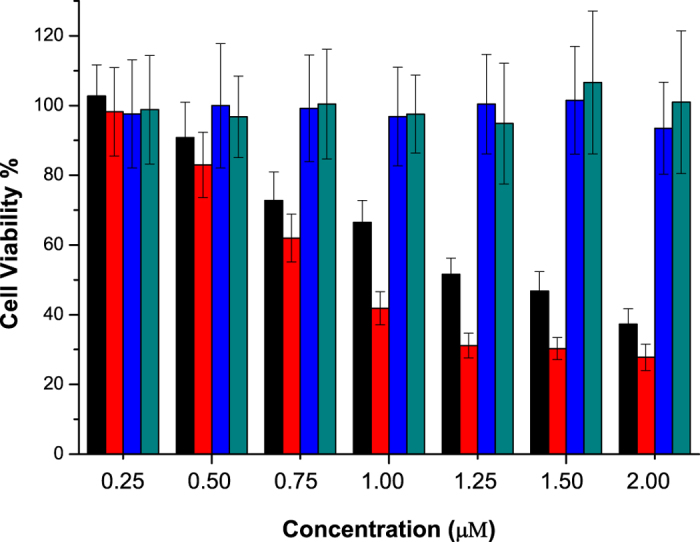
Effects of **FPB** and F16 on cell proliferation (5 days) at different concentrations. Black, effect of **FPB** on SGC-7901 cells proliferation. Red, effect of F16 on SGC-7901 cells proliferation. Blue, effect of **FPB** on NIH/3T3 cells proliferation. Cyan, effect of F16 on NIH/3T3 cells proliferation. Data are presented as the mean ± SD of two independent experiments.

Information on cell proliferation is one of the most the important indicators in the evaluation of drug effect and physiological research. The MTT cell proliferation assay has already been demonstrated to be a rapid, versatile, quantitative, and highly reproducible colorimetric assay for determining viable cell number in proliferation^28^,^29^. The effects of **FPB** on cell proliferation were measured by MTT cell proliferation assay using SGC-7901 cells and 3T3 cells. The effect of F16 was also studied for comparison. As shown in Fig. 3, the proliferation of SGC-7901 cells was significantly inhibited by 2 μM **FPB** or F16 upon 5 days of exposure while 3T3 cells proliferation was not affected under the same conditions. Compared with F16, **FPB** shows a little weaker efficiency on inhibition of cancer cell proliferation at low concentration. At a concentration of 2 μM, the difference is approximately 10%. Based on these results, we can conclude that **FPB** has a similar selectivity and anti-proliferative activity as F16.

### Mitochondria are the major concentrating organelle of FPB in the cell

**Figure 4:**
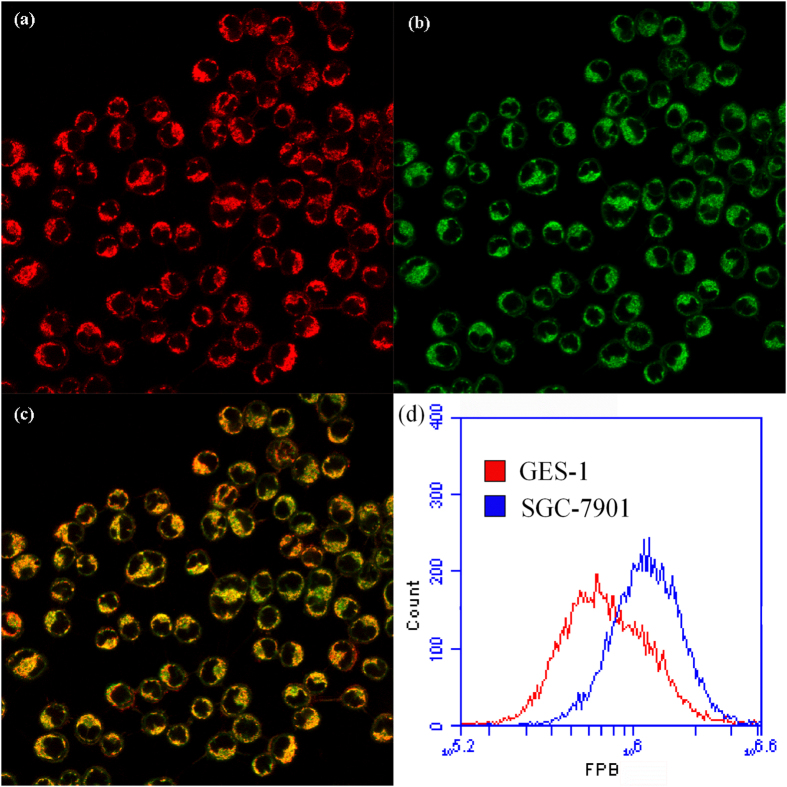
(**a**) Fluorescence imaging of Mito Tracker Red (0.5 μM, *λ*_em_=598 nm) in SGC-7901 cells. (**b**) Fluorescence imaging of **FPB** (0.5 μM, *λ*_em_ = 514 nm) in SGC-7901 cells. (**c**) Overlapped image of (**a**) and (**b**). (**d**) Flow cytometry analysis of **FPB** uptake content in SGC-7901 cells and GES-1 cells.

To access the intracellular distribution of **FPB** in SGC-7901 cells, we performed a colocalization experiment using Nikon A1 Confocal laser scanning microscope. The cells were first incubated with **FPB** in the culture medium for 24 h, and then stained with Mito Tracker Red, a commercial mitochondrial dye. Figure 4b directly suggested that **FPB** display intriguing fluorescent property in SGC-7901 cells and could be directly visualized in living cells. From a contrasting merged image of **FPB** and Mito Tracker Red staining, we can observe that the **FPB** fluorescence perfectly overlapped with the mitochondria staining (Fig. 4c), which indicated that the mitochondria were the major concentrating organelle of **FPB** in the cell. Moreover, the optical properties of **FPB** were investigated in aqueous solution (Fig. S3 (SI)). **FPB** exhibits strong and narrow emission at 514 nm (full-width at half-maximum = 30 nm) and the fluorescence quantum yield is 20.2% (using fluorescein in ethanol as the reference). The remarkable optical properties allowed the direct visualization of **FPB** in cells. The results strongly demonstrate the excellent mitochondrial targeting and subcellular imaging capability of **FPB** in living carcinoma cells.

### Higher cellular uptake in cancer cells

We then investigated the cellular uptake of **FPB** for SGC-7901 and GES-1 cells with flow cytometry. Cultured cell lines were incubated with 0.5 μM **FPB** for 24 h, and then we studied the uptake content of **FPB** by its intrinsic fluorescence. As shown in Fig. 4d, the **FPB** uptake capability of SGC-7901 cells is significantly greater than that of GES-1 cells. These results suggest that **FPB** could selectively accumulate in cancer cells. The preferential accumulation of **FPB** in cancer cell lines accounted for higher toxicity to cancer cells than to normal cells.

### FPB induces cell apoptosis

**Figure 5:**
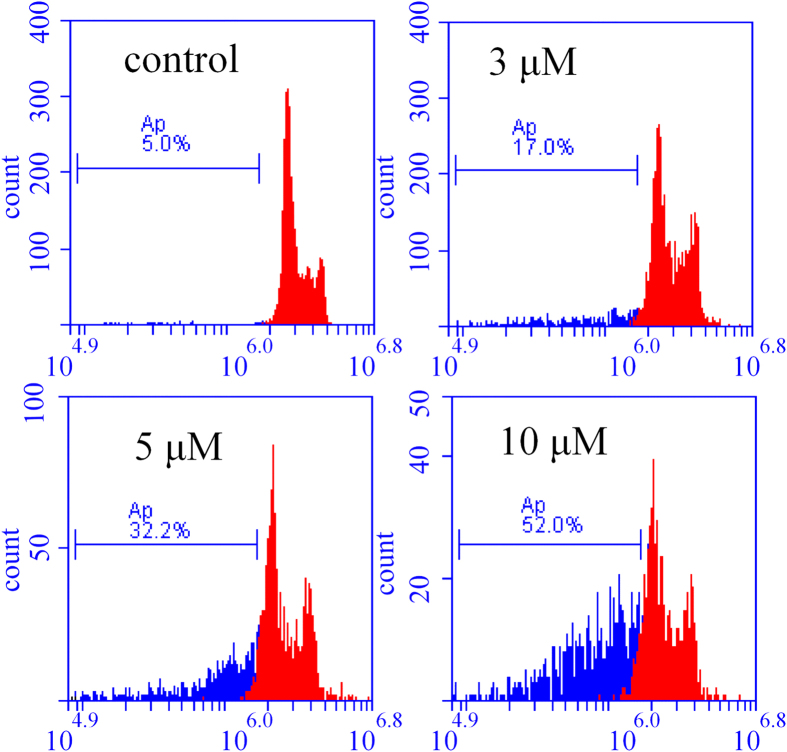
DNA fluorescence histograms of PI-stained SGC-7901 cells treated with FPB of different concentrations. The values in figure represented the percentage of apoptosis.

To further study the anticancer mechanism of **FPB**, we performed apoptosis assay of SGC-7901 cells after incubation with **FPB** for 48 h by flow cytometry. Cells were stained with propidium (PI) dye for nucleic acid staining. Because the apoptotic cells are characterized by DNA fragmentation at the internucleosomal section and consequently loss of nuclear DNA content, they display a broad hypodiploid (sub-G1) peak after stained with PI and analysed with flow cytometer^30^. As shown in Fig. 5, flow cytomeric analysis of PI-stained apoptotic cells shows a distinctive broad peak of hypodiploid particles, clearly different from the diploid DNA peak of normal cells. The proportion of apoptotic cells increased in a dose-dependent manner. At 10 μM of **FPB**, the percentage of apoptotic cells is over 50% (Fig. 5d). Moreover, contrast cell volume behaviours (swelling vs. shrinkage) are considered as important markers to identify apoptosis and necrosis^31^. Thus laser light scatter characteristics of cell morphology could also be used to reflect cell apoptosis^32^. Markedly decrease in intensity of forward light scatter (FSC) signal and increase in intensity of side scatter (SSC) signal were observed for cells after treated with **FPB** (Fig. S6 (SI)), reflecting the cell volume shrinkage and condensation of nucleus and cytoplasm, which is a typical morphological feature of apoptotic cells. The reduction in cell volume is also illustrated in direct phase contrast images (Fig. 2c,d). Combining the DNA fragmentation and cell morphological features, we concluded that **FPB** could effectively induce cell apoptosis to kill cancer cells.

### FPB induce dissipation of *ΔΨ*_*m*_ and increase in ROS level

**Figure 6:**
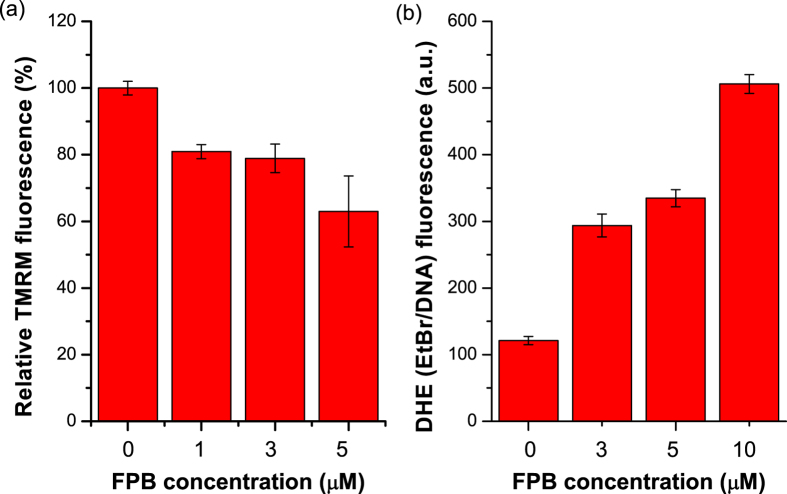
Effects of FPB on mitochondrial membrane potential and ROS level. (**a**) Mitochondrial membrane potential is reflected as mean fluorescence intensity of TMRM determined by flow cytometry. (**b**) Flow cytometry analysis of ethidium bromide-DNA fluorescence resulting from DHE oxidation as a measure of ROS levels. Data are presented as the mean ± SD of three independent experiments.

Next, we analysed physiological alterations of mitochondria in cancer cells treated by **FPB**. A decrease in *ΔΨ*_*m*_ is a hallmark of cells that precede apoptosis through the mitochondria insults^33^. The dissipation of *ΔΨ*_*m*_ could be detected by the fluorescent probe tetramethylrhodamine methyl ester (TMRM) which accumulates in the mitochondrial matrix in proportion to *ΔΨm*^34^. A representative flow cytometric plot reflecting the effect of **FPB** on SGC-7901 cells after incubation for 48 h is shown in Fig. S7 and mean fluorescence intensity of TMRM was reflected in Fig. 6a. As shown in Fig. 6a, treatment of SGC-7901 cells with **FPB** could induce dissipation of *ΔΨ*_*m*_ in a dose-dependent manner. As apoptosis is often accompanied by a decrease in *ΔΨ*_*m*_, this result further confirms that **FPB** could induce cell apoptosis.

Reactive oxygen species (ROS), such as hydrogen peroxide (H_2_O_2_) and superoxide anion (O_2_^−^), are generated as a by-product of respiration and play an important role as a messenger in cellular signal transduction. ROS are expected to be more abundant during apoptosis and in response to mitochondrial dysfunction^35^. We therefore measured the intracellular ROS level of **FPB** treated cancer cells using a fluorescent probe, dihydroethidium (DHE). The non-fluorescent DHE could penetrate cells freely and interact with O_2_^−^ to form the membrane-impermeant ethidium cation, which becomes fluorescent upon intercalating DNA, thus the fluorescence intensity of ethidium-DNA can indicate the level of intracellular ROS^36^. As shown in Fig. 6, intracellular superoxide anion concentration increased over 4 times with 10 μM of **FPB** in SGC-7901 cells. The significant increase in O_2_^−^ level with **FPB** treatment indicated that the mitochondria in cancer cells are damaged or in serious disorders.

## Discussion

Recently, a lot of attention has been drawn to mitochondria as potential targets of anticancer therapy because of their crucial involvement in cell death^2^,^3^,^10^. The **FPB** drug we reported here could specifically target the mitochondria and will attract great interest. Driving by the mitochondrial membrane potential, **FPB** could accumulate in cancer cell mitochondrial and affect the function of mitochondria. Our data confirmed that **FPB** could efficiently inhibit cancer cell proliferation and induce cell apoptosis. The selectivity and imaging capabilities make it highly attractive in cancer diagnosis and therapy.

Many established mitochondrial-targeted drugs have already achieved important breakthrough in the management of malignancies. α-TOS is a redox-inactive vitamin E analogue that selectively triggers mitochondrial apoptosis in tumour cells and suppresses the growth of many types of carcinomas in preclinical models^37^. A peptide trivalent arsenical, 4-(N-(S-glutathionylacetyl)amino) phenylarsenoxide (GSAO), is shown to inactivate the adenine nucleotide translocator (ANT) and cause apoptosis in proliferating but not growth-quiescent endothelial cells^35^. 3-bromopyruvate (3-BrPA) is a promising antitumor agent which has recently been accepted for a Phase I clinical trial in liver cancer and has been reported to cause a covalent modification of HKII protein and directly triggered its dissociation from mitochondria, leading to a specific release of apoptosis-inducing factor (AIF) from the mitochondria to cytosol and eventual cell death^38^,^39^. Similar to these well-established mitochondrial-targeted drugs, **FPB** could also selectively target tumour cells and cause increase in superoxide levels, mitochondrial depolarization and apoptosis in proliferating. The lower *IC*_50_ values of **FPB** towards cancer cells indicate that **FPB** has better antitumor efficiency according to the anticancer cytotoxic action of these drugs. More importantly, **FPB** exhibits excellent fluorescent property and can achieve simultaneously treatment and visualisation of cancer cells which would be beneficial in clinical treatment of malignancies.

In summary, we have synthesised and characterised a F16-BODIPY conjugate and evaluated its *in vitro* anticancer activity and organelle imaging capability. The conjugate could selectively accumulate in cancer cell mitochondria and induce cell death. The *IC*_50_ values of the conjugate toward cancer cells are 2 ∼ 20 times smaller than that toward normal cells. The selectivity of the conjugate is mainly due to its different uptake in cancer and normal cells. Excellent fluorescent property enables its use for subcellular imaging simultaneously. DNA staining assay and cellular morphology images suggest that the conjugate could effectively induce cell apoptosis. **FPB** treatment causes a concentration-dependent increase in mitochondrial depolarization and superoxide level in cancer cells. Our findings suggest that **FPB** could be used as a bifunctional anticancer agent that enables selective anticancer activity and efficient subcellular imaging simultaneously.

## Methods

### Synthesis of compound C1

A mixture of pyridnecarboxaldehyde (0.30 mL, 3.0 mmol), N-butyl phosphorus (0.74 mL, 3.0 mmol), gramine (348 mg, 2.0 mmol), and 5.0 mL acetonitrile was stirred at 60 °C for 24 h. The mixture was concentrated under vacuum, and the oil product was purified by silica chromatography with hexanes/EtOAc (1:1 V/V) to give C1 (181 mg, yield 41%). ^1^H NMR (400 MHz, DMSO-d6): δ = 7.03–7.19 (m, 3H), 7.42–7.52 (m, 3H), 7.69–7.74 (d, 2H), 8.03 (d, 1H), 8.45 (d, 2H), 11.50 (s, 1H); HRMS: Exact mass calculated for C_15_H_12_N_2_ (M^+^) 221.1073; Found: 221.1079.

### Synthesis of compound C2

The 1-bromo-4-iodobenzene (200 mg, 0.71 mmol) was added to a suspension of C1 (200 mg, 0.9 mmol), K_2_CO_3_ (105 mg, 0.76 mmol), CuI (12 mg, 0.063 mmol), piperidine-2-carboxylic acid (15 mg, 0.12 mmol) in dry DMF (5 mL) and stirred at 110 °C for 24 h. The product was separated by silica chromatography with hexanes/EtOAc (1:1 V/V) and recrystallized with EtOAc/H_2_O to give C2 (121 mg, yield 50%). C2 is a yellow powder and soluble in water. ^1^H NMR (400 MHz, DMSO-d6): δ = 7.15–7.28 (m, 3H), 7.42–7.62 (m, 5H), 7.72–7.78 (m, 2H), 7.95 (d, 1H), 8.05 (d, 1H), 8.15 (m, 1H), 8.50 (d, 2H); HRMS: Exact mass calculated for C_21_H_15_BrN_2_ (M^+^) 337.0473; Found: 337.0470.

### Synthesis of compound C3

A mixture of C2 (225 mg, 0.67 mmol), trimethyl silyl acetylene (88 mg, 0.9 mmol), Pd(PPh_3_)_4_ (20 mg, 0.05 mmol) and CuI (12 mg, 0.063 mmol) was added to 6 mL THF/Et_3_N (5:1, V/V) and stirred at 45 °C overnight. The product was purified by silica chromatography with hexanes/EtOAc (1:1 V/V) to give yellow oily C3 (194 mg, yield 90%). ^1^H NMR (400 MHz, DMSO-d6): δ = 4.31 (s, 1H), 7.15–7.25 (m, 3H), 7.55–7.78 (m, 8H), 8.10–8.14 (m, 2H), 8.50 (d, 1H), 8.15 (m, 1H), 8.50 (d, 2H); HRMS: Exact mass calculated for C_23_H_16_N_2_ (M^+^) 321.1386; Found: 321.1390.

### Synthesis of compound BODIPY-I

p-Iodobenzaldehyde (2.32 g, 10 mmol), and 2,4-dimethylpyrrole (1.90 g, 20 mmol) were dissolved in 250 mL of dry CH_2_Cl_2_. 1 mL of TFA was added to the reaction mixture, the mixture was stirred for 3 h at room temperature under Argon atmosphere. DDQ (2.26 g, 10 mmol) was added. After stirring for 30 min, triethylamine (20.0 mL) and BF_3_•OEt_2_ (20.0 mL) were added and stirred overnight. The organic product was purified by column chromatography on silica gel using hexane/DCM (70/30, v/v) to obtain an orange crystalline solid (0.2 g, yield 4.4%). ^1^H NMR (400 MHz, CDCl3): δ = 1.41 (s, 6H), 2.25 (s, 6H), 5.99 (s, 2H), 7.04 (d, 2H), 7.84 (d, 2H).

### Synthesis of compound C4

C4 was prepared from C3 (100 mg, 0.31 mmol) and BODIPY-I (215 mg, 0.50 mmol) as the previous synthesis procedure for C3 in 33% yield (65 mg). ^1^H NMR (400 MHz, DMSO-d6): δ = 1.42 (s, 6H), 2.47 (s, 6H), 6.22 (s, 2H), 7.23–7.35 (m, 3H), 7.48 (d, 2H), 7.59 (d, 2H), 7.68–7.86 (m, 6H), 8.18 (m, 2H), 8.33 (s, 2H), 8.52 (d, 2H).

### Synthesis of compound FPB

C4 (10.0 mg, 0.016 mmol) and CH_3_I (10 μL) were added to 3 mL dry DMF at room temperature and stirred overnight. DMF was removed and the residues were recrystallized in CHCl_3_ and n-hexane to obtain C5 (11.0 mg, yield 88%). ^1^H NMR (400 MHz, DMSO-d6): δ = 1.43 (s, 6H), 2.48 (s, 6H), 4.23 (s, 3H), 6.23 (s, 2H), 7.41 (dd, J = 6.2, 3.0 Hz, 2H), 7.57–7.45 (m, 3H), 7.71 (dd, J = 6.5, 2.7Hz, 1H), 7.80 (dd, J = 8.4, 4.8 Hz, 4H), 7.88 (d, J = 8.6 Hz, 2H), 8.21 (d, J = 6.9 Hz, 2H), 8.35–8.26 (m, 2H), 8.36 (s, 1H), 8.78 (d, J = 6.9 Hz, 2H); HRMS: Exact mass calculated for C_42_H_36_BF_2_N_4_^+^(M^+^) 657.3003; Found: 657.3068.

### Synthesis of compound F16-Ph-Ace

F16-Ph-Ace was prepared from C3 (50 mg, 0.16 mmol) and CH_3_I (10 μL) as the previous synthesis procedure for C5 with a yield of 92% (68 mg). ^1^H NMR (400 MHz, DMSO-d6): δ = 4.22 (s, 3H), 4.38 (s, 1H), 7.37–7.50 (m, 3H), 7.64–7.74 (m, 5H), 8.18–8.30 (m, 5H), 8.76 (d, 2H); HRMS: Exact mass calculated for C_24_H_19_N_2_ (M^+^) 335.1542; Found: 335.1549.

### Photophyscial Studies

Fluorescence quantum yields (*Φ*_F_) were measured according to the equation:
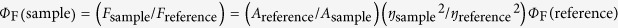
Where F, A, and *ŋ* are the integrated fluorescence area (Ex = 501 nm, area under the emission peak), the absorbance at the excitation wavelength (501 nm), and the relative index of the solvent, respectively. Fluorescein in ethanol was used as the reference with a value for *Φ*_F_ = 0.92^40^.

### Cytotoxicity assay

Six kinds of cells were cultured in DMEM medium containing 10% fetal bovine serum (FBS, Gibco) in a humidified atmosphere containing 5% CO_2_ at 37 °C. The cells were seeded onto 96-well plates at 10^4^ cells per well and incubated for 24 h. **FPB** with varying concentrations were respectively added into the cells followed by further culture of 48 h and the culture media were discarded and a MTT solution in PBS (0.5 mg/mL, 100 μL per well) were added to each well followed by incubation for 4 h. The supernatant was abandoned and 150 μL DMSO was added into each well. The plate was then incubated at 40 °C and shaken for 10 min. The absorbance value at 570 nm at each well was recorded by a microplate reader. The cell viability rate (VR) was calculated according to the following equation:
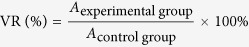
where the control group was carried out in the absence of **FPB** drug. The survival curves plotted as a function of concentration of **FPB** and cell viability rate (%). The *IC*_50_ values were determined as the concentration that cause 50% inhibition of cell viability and were analyzed by the statistic software Statistical Product and Service Solutions (SPSS Version 13.0).

### Phase contrast imaging

The SGC-7901 cells suspension were plated in 35 mm dishes and incubated in 1 mL DMEM medium for 24 h. The cells were washed once with PBS and then grown in 1 mL DMEM medium with and without **FPB** (5 μM) for 48 h. Phase contrast bright-field images were taken using fluorescence microscopy.

### The MTT Cell Proliferation Assay

The SGC-7901 cells and NIH/3T3 cells were seeded onto 96-well plates and incubated for 24 h. On the following day, different concentration of **FPB** or F16 was added to the cells and incubated for 5 days. The ability of viable cells was determined by MTT method as described above.

### Confocal Laser Scanning Microscopy Measurements

SGC-7901 cells were seeded onto 35 mm dishes and incubated for 24 h for attachment. Cells were then treated with **FPB** (0.5 μM) for 24 h. Mitotracker Red (0.5 μM) was used to stain the cells for 15 min. The cells were then examined by confocal laser scanning microscopy.

### Cellular uptake

SGC-7901 cells and GES-1 cells suspension were seeded onto 6-well plates and adhered to the walls overnight. After incubation with **FPB** (0.5 μM) for 24 h, the cells were washed 3 times with PBS, digested with trypsin, resuspended in PBS and then analyzed with BD ACCURI C6 flow cytometer by detecting the fluorescence of **FPB**.

### PI-staining cell apoptosis assay

SGC-7901 cells were incubated in DMEM medium with **FPB** (0, 3, 5, 10 μM) for 48 h. Then the cells were harvested, centrifuged, and suspended in PBS, and fixed by 70% (v/v) cold ethanol solution at 4 °C overnight. Then the cells supernatant (ethanol solution) was removed. The cells were washed with PBS and then resuspended in DNA staining solution (10 mL PBS containing 0.1% sodium citrate, 0.1% Triton X-100, 2 mg RNase and 500 μg propidium iodide, pH = 7.4). After incubating with PI solution for 30 min at 4 °C, the cells were analyzed with BD ACCURI C6 flow cytometer to record red fluorescence of PI.

### Determination of mitochondrial membrane potential

Changes in mitochondrial membrane potential of **FPB** treated SGC-7901 cells was obtained by flow cytometry, using the potentiometric probe tetramethylrhodamine methyl ester (TMRM). SGC-7901 cells were grown with **FPB** (0, 1, 3, 5 μM) for 48 h and resuspended in PBS. TMRM were added to a final concentration of 200 nM. After incubated for 20 min, the cells were analysed by flow cytometry.

### Superoxide assay

SGC-7901 cells were grown with **FPB** (0, 3, 5, 10 μM) for 48 h and resuspended. To measure superoxide levels, the cells were incubated with 4 μM dihydroethidium for 15 min at room temperature, then immediately assessed by flow cytometry.

## Additional Information

**How to cite this article**: He, H. *et al.* A novel bifunctional mitochondria-targeted anticancer agent with high selectivity for cancer cells. *Sci. Rep.* **5**, 13543; doi: 10.1038/srep13543 (2015).

## Supplementary Material

Supplementary Information
