# Supplementary material for: A novel bifunctional mitochondria-targeted anticancer agent with high selectivity for cancer cells
Source: Sci Rep. 2015 Sep 4;5:13543. doi: 10.1038/srep13543 (PMC4559806; doi:10.1038/srep13543)
Supplement: Supplementary Information [file srep13543-s1.pdf]

Supporting Information

A novel bifunctional mitochondria-targeted anticancer agent with high selectivity for cancer cells

Huan He, Dong-Wei Li, Li-Yun Yang, Li Fu, Xun-Jin Zhu, Wai-Kwok Wong, Feng-Lei Jiang*, and Yi Liu*

Fig. S1 Synthesis of BODIPY-I

Fig. S2 Synthesis of F16-Ph-Ace

Fig. S3 Excitation spectra and fluorescence spectra of **FPB**

Fig. S4 Fluorescent quantum yield of **FPB**

Fig. S5 Cytotoxic effects of **FPB**

Fig. S6 Flow cytometric analysis of **FPB** on SGC-7901 cells

Fig. S7 Effects of **FPB** on $\Delta\Psi_m$ and ROS level

Fig. S8 ^{1}H NMR spectrum of **FPB**

Fig. S9 HRMS spectrum of **FPB**

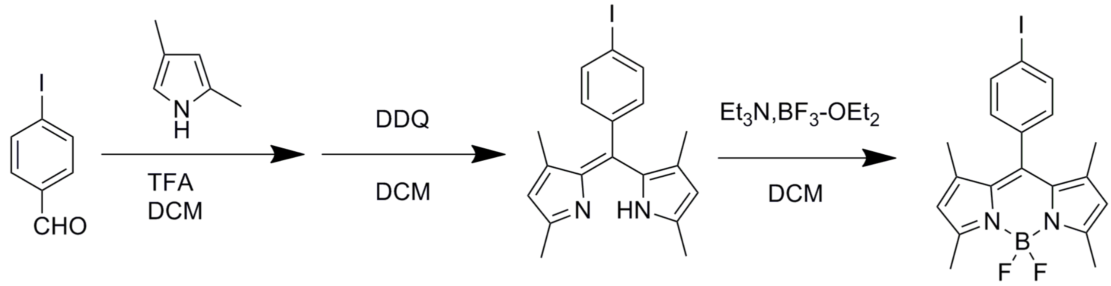

Fig. S1. Synthesis of BODIPY-I

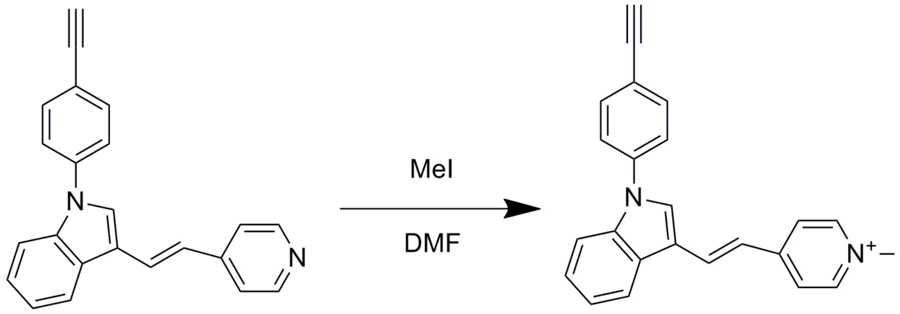

C 3

F16-Ph-Ace

Fig. S2. Synthesis of F16-Ph-Ace

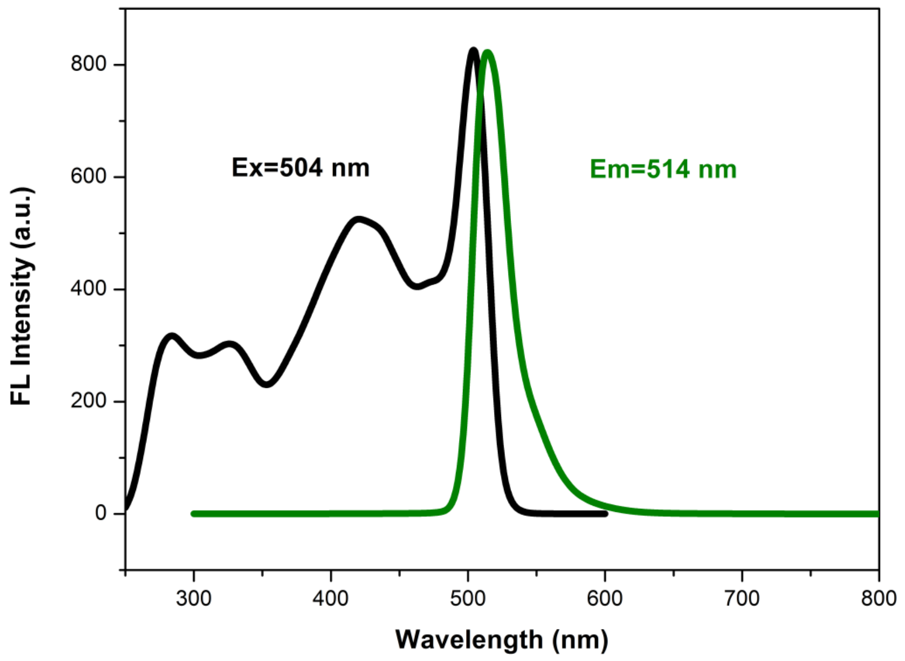

Fig. S3. Excitation spectra (black) and fluorescence (green) spectra of **FPB**

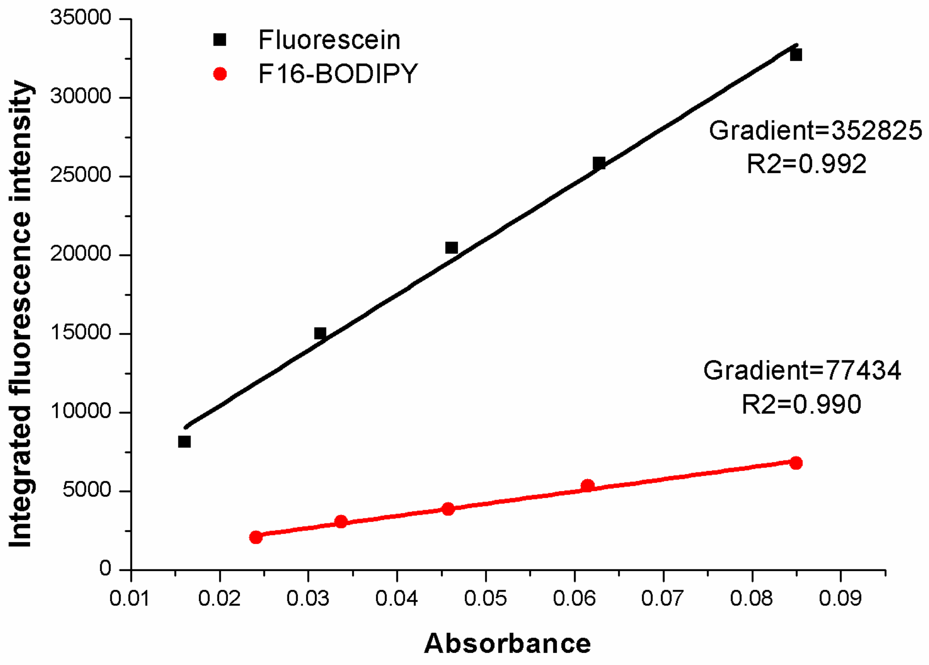

Fig. S4. Fluorescent quantum yield of **FPB** (using fluorescein in ethanol as the reference)

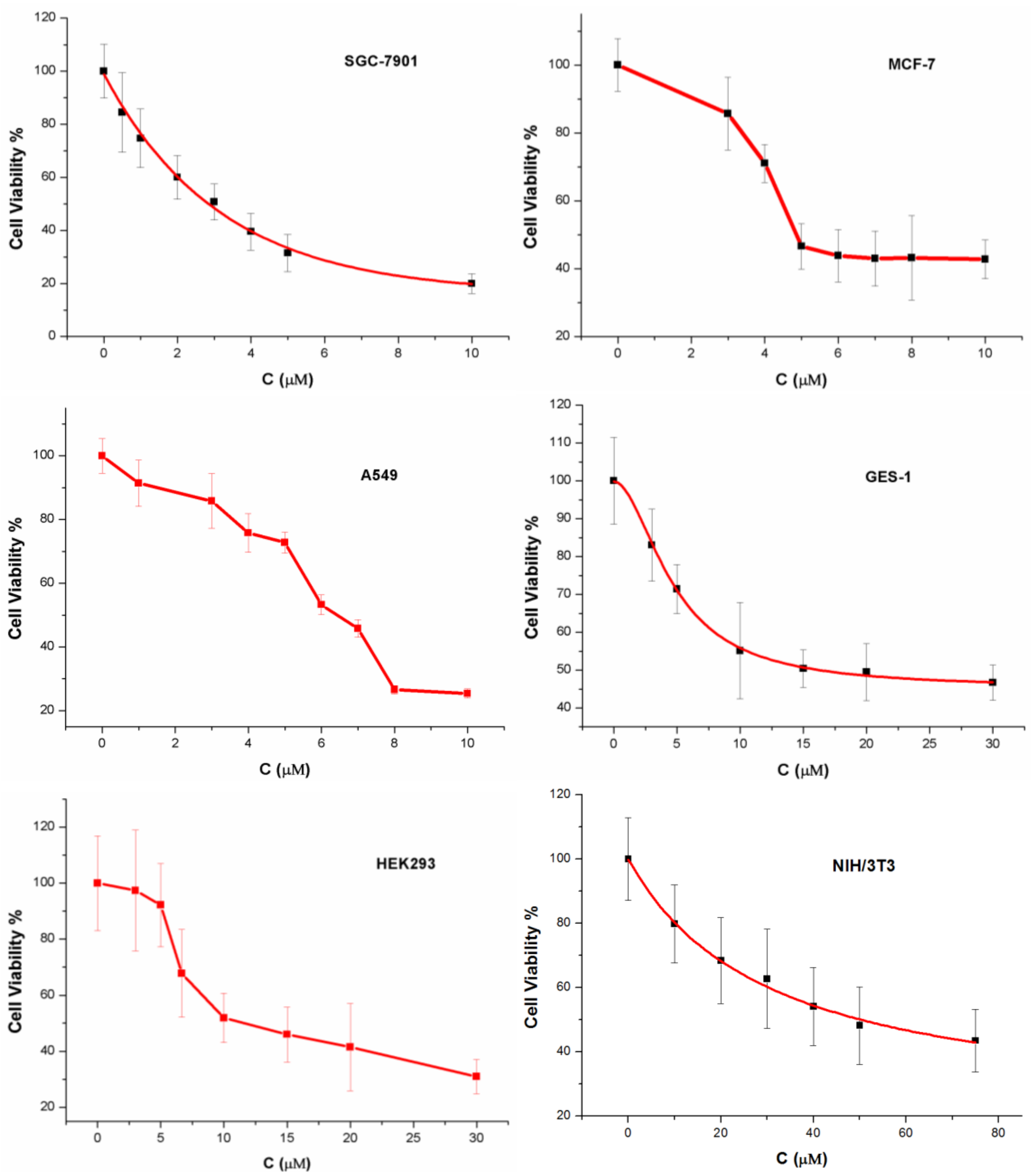

Fig. S5. Cytotoxic effects of **FPB** towards SGC-7901, MCF-7, A549, GES-1, HEK293 and NIH/3T3 cells using the MTT assays

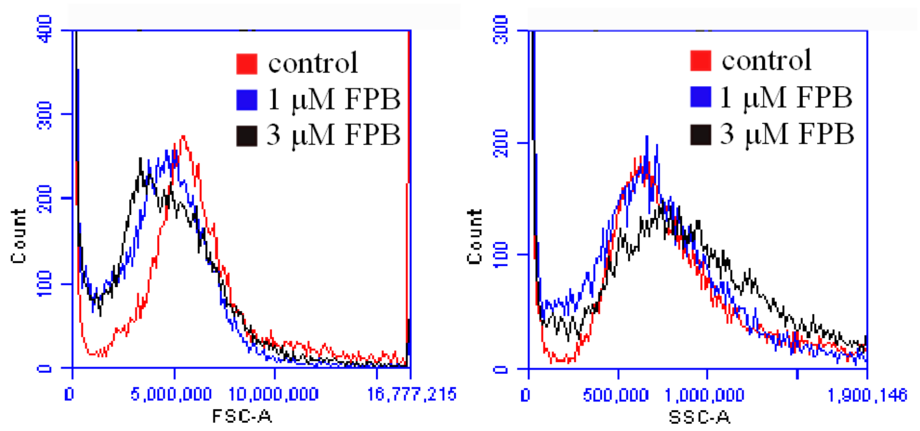

Fig. S6. Representative flow cytometric plots reflecting the effect of **FPB** on SGC-7901 cells

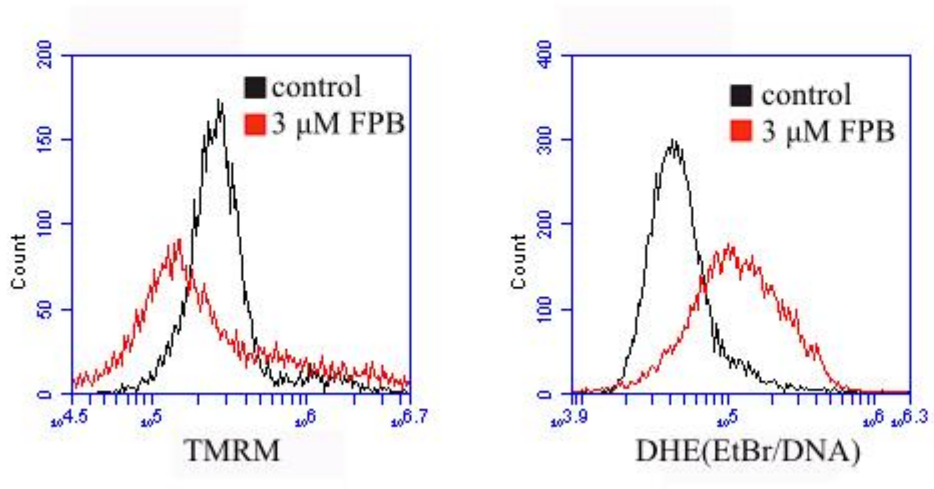

Fig. S7. Representative flow cytometric plots reflecting the effects of **FPB** on mitochondrial membrane potential and ROS levels.

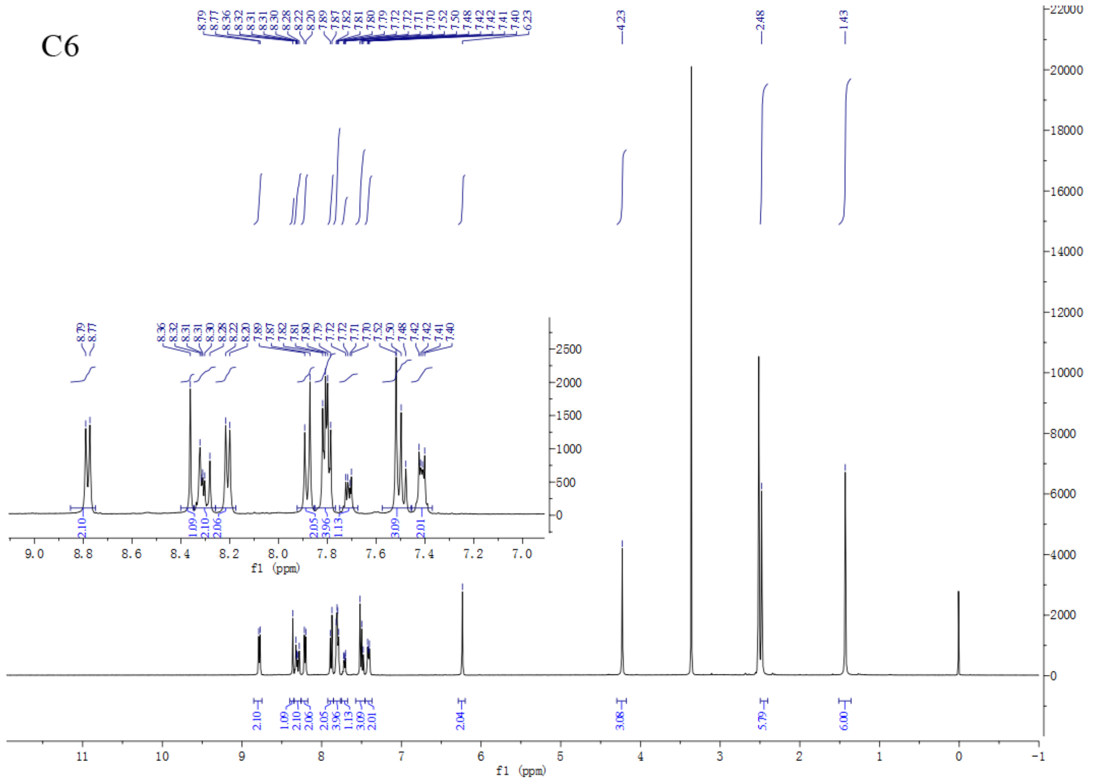

Fig. S8 ^{1}H NMR spectrum of **FPB** in DMSO-d_6

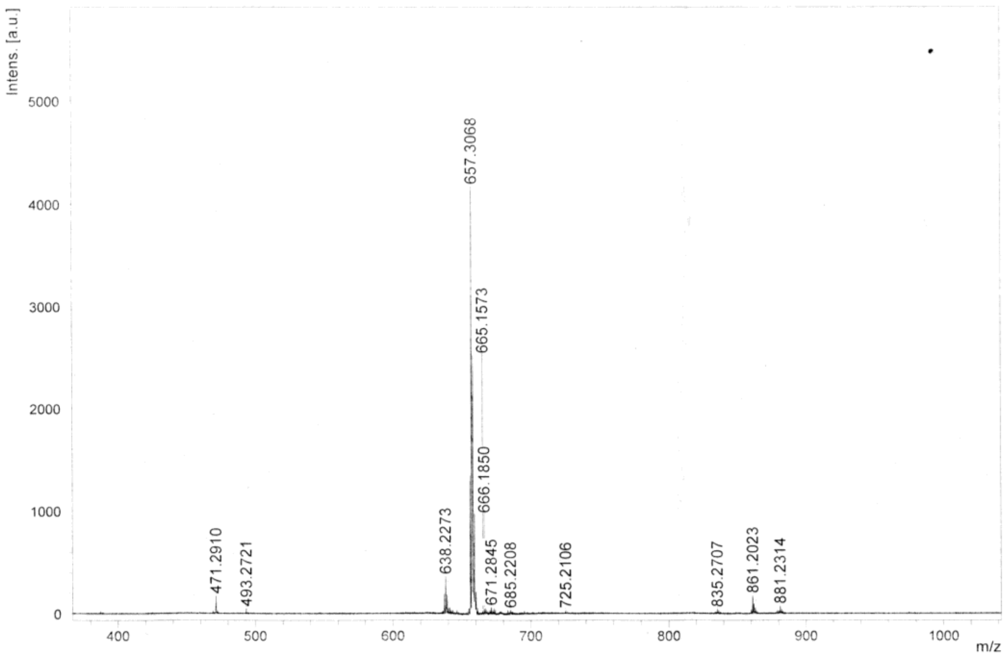

Fig. S9. HRMS spectrum of **FPB**
